# Succinate dehydrogenase inhibition leads to epithelial-mesenchymal transition and reprogrammed carbon metabolism

**DOI:** 10.1186/2049-3002-2-21

**Published:** 2014-12-15

**Authors:** Paul-Joseph P Aspuria, Sophia Y Lunt, Leif Väremo, Laurent Vergnes, Maricel Gozo, Jessica A Beach, Brenda Salumbides, Karen Reue, W Ruprecht Wiedemeyer, Jens Nielsen, Beth Y Karlan, Sandra Orsulic

**Affiliations:** Women’s Cancer Program, Samuel Oschin Comprehensive Cancer Institute, Cedars-Sinai Medical Center, 8700 Beverly Blvd, Los Angeles, CA 90048 USA; Department of Physiology, Michigan State University, East Lansing, MI 48824 USA; Koch Institute for Integrative Cancer Research, Massachusetts Institute of Technology, Cambridge, MA 02139 USA; Systems and Synthetic Biology, Department of Chemical and Biological Engineering, Chalmers University of Technology, Gothenburg, 41296 Sweden; Department of Human Genetics, David Geffen School of Medicine, University of California Los Angeles, Los Angeles, CA 90095 USA; Graduate Program in Biomedical Science and Translational Medicine, Cedars-Sinai Medical Center, Los Angeles, CA 90048 USA; Department of Obstetrics and Gynecology, David Geffen School of Medicine, University of California Los Angeles, Los Angeles, CA 90095 USA

**Keywords:** Succinate dehydrogenase, SDH, Ovarian cancer, EMT, Carbon metabolism, Epigenetics

## Abstract

**Background:**

Succinate dehydrogenase (SDH) is a mitochondrial metabolic enzyme complex involved in both the electron transport chain and the citric acid cycle. SDH mutations resulting in enzymatic dysfunction have been found to be a predisposing factor in various hereditary cancers. Therefore, SDH has been implicated as a tumor suppressor.

**Results:**

We identified that dysregulation of SDH components also occurs in serous ovarian cancer, particularly the SDH subunit SDHB. Targeted knockdown of *Sdhb* in mouse ovarian cancer cells resulted in enhanced proliferation and an epithelial-to-mesenchymal transition (EMT). Bioinformatics analysis revealed that decreased SDHB expression leads to a transcriptional upregulation of genes involved in metabolic networks affecting histone methylation. We confirmed that *Sdhb* knockdown leads to a hypermethylated epigenome that is sufficient to promote EMT. Metabolically, the loss of *Sdhb* resulted in reprogrammed carbon source utilization and mitochondrial dysfunction. This altered metabolic state of *Sdhb* knockdown cells rendered them hypersensitive to energy stress.

**Conclusions:**

These data illustrate how SDH dysfunction alters the epigenetic and metabolic landscape in ovarian cancer. By analyzing the involvement of this enzyme in transcriptional and metabolic networks, we find a metabolic Achilles’ heel that can be exploited therapeutically. Analyses of this type provide an understanding how specific perturbations in cancer metabolism may lead to novel anticancer strategies.

**Electronic supplementary material:**

The online version of this article (doi:10.1186/2049-3002-2-21) contains supplementary material, which is available to authorized users.

## Background

Ovarian cancer is the most lethal gynecological malignancy in the United States, resulting in approximately 22,000 new diagnoses and 14,000 deaths in 2013 [1]. The prognosis of ovarian cancer is poor and has seen limited improvement in the past few decades with a 5-year survival rate of roughly 44% [2]. High-grade serous ovarian cancer is the most common and most lethal subtype of ovarian cancer, contributing to >70% of deaths from ovarian cancer [3]. Integrative large-scale analyses of patient tumors with serous histology have revealed that the disease is highly heterogeneous but can be clustered into several molecular subtypes with different clinical outcomes [4, 5]. One of the poor prognosis subtypes contains an epithelial-mesenchymal transition (EMT) signature [5]. Therefore, understanding and characterizing EMT in ovarian cancer is of great importance.

EMT is a biological process that converts epithelial cells to motile, mesenchymal cells [6]. It is largely defined by the loss of plasma membrane E-cadherin and conversion from a cobblestone-like morphology to a more spindle-shaped and less adherent state. It was initially characterized as a fundamental event in morphogenesis during embryonic tissue and organ development [7]. However, emerging evidence suggests that cancer cells utilize the EMT program to enhance invasiveness, increase migratory ability, and acquire stem-like characteristics such as chemoresistance. EMT reprogramming involves several layers of regulation and has been the subject of intense research, especially in the context of cancer [6]. One area that has not been extensively studied is the role of metabolic enzymes in the regulation and maintenance of EMT.

Alterations in metabolism have been implicated in cancer, with the main focus on the Warburg effect, a phenomenon in which cancer cells upregulate glycolysis and lactate production while decreasing glucose contribution to the citric acid (TCA) cycle in the mitochondria, even in the presence of sufficient oxygen [8–10]. However, mitochondrial metabolism is also important for cancer proliferation. For example, mutations in the mitochondrial enzyme succinate dehydrogenase (SDH) have been reported in a wide variety of cancers [11, 12]. Also known as mitochondrial respiratory Complex II, SDH is the only enzyme to participate in both the TCA cycle and electron transport chain (ETC.). In the TCA cycle, SDH catalyzes the oxidation reaction where succinate is converted to fumarate, which is coupled to the reduction of ubiquinone to ubiquinol in the ETC. [12]. SDH is a holoenzyme consisting of four essential subunits: SDHA (a flavoprotein), SDHB (iron-sulfur protein), and two membrane anchor units SDHC and SDHD. SDH assembly requires two factors, SDHAF1 and SDHAF2 [13], while its function is influenced by the deacetylase activity of SIRT3 [14]. *SDHx* loss-of-function mutations have been found to predispose individuals to hereditary pheochromocytoma (PCC), paraganglioma (PGL), gastrointestinal stromal tumor (GIST), and renal cell carcinoma [12]. Interestingly, it has been shown that *SDHx* mutations are associated with hallmarks of EMT in PCC and PGL as a result of alterations to the epigenome [15, 16]. Furthermore, the SDH regulators SDHAF1 and SIRT3 have been identified as tumor suppressor genes in lung and breast cancer, respectively [14, 17]. This has led to the definition of SDH as a mitochondrial tumor suppressor and a key player in cancer cell differentiation.

Ovarian cancer is characterized by a remarkable degree of genomic disarray but extremely low frequency of recurrent genomic alterations [4]. The dearth of common therapeutic targets in ovarian cancer samples highlights the need for individualized therapy or different approaches to identifying patient groups with common characteristics that can be therapeutically exploited. Interestingly, succinate was identified as a potential metabolic urinary biomarker for ovarian cancer [18], indicating that altered SDH activity may be important in ovarian cancer progression.

Here, we investigate whether alterations in SDH function also impact ovarian cancer biology. We find that dysregulation of SDH members occurs frequently in serous ovarian cancer. Utilizing a mouse ovarian cancer cell line as a model system, we find that knockdown of one of the SDH subunits, *Sdhb*, increases aggressiveness and results in EMT. *Sdhb* knockdown cells were found to have a hypermethylated epigenome whereby increased H3K27 methylation was sufficient to induce EMT. Using a metabolomic approach to investigate the metabolic effects of decreased SDH activity, we found that *Sdhb* knockdown leads to altered glucose and glutamine utilization through central carbon metabolism as well as mitochondrial dysfunction. These data demonstrate the metabolic and epigenetic consequences of decreased activity of a mitochondrial metabolic enzyme in ovarian cancer.

## Methods

### Plasmids and reagents

pLKO.1-based short hairpin constructs specific for mouse *Sdhb* and human *SDHB*, as well as scrambled control sequences, were obtained from Sigma-Aldrich (St. Louis, MO, USA). shRNA sequences are listed in Additional file 1: Table S1. GSK-J1 and GSK-J4 were obtained from Tocris Bioscience (Bristol, Avon, UK). Metformin, p-iodonitrotetrazolium, oligomycin, carbonyl cyanide 4-(trifluoromethoxy)phenylhydrazone chloride (FCCP), and rotenone-myxothiazol were purchased from Sigma-Aldrich.

### Cell lines

C1 mouse ovarian cancer cells were generated as previously described [19–21]. HEY cells were obtained from American Type Culture Collection (ATCC). Both cell lines were grown in Dulbecco’s modified Eagle’s medium (DMEM) supplemented with 10% FBS unless otherwise indicated.

### Lentiviral-mediated gene knockdown

pLKO.1-sh1-*Sdhb*, pLKO.1-sh2-*Sdhb*, pLKO.1-sh1-*SDHB*, pLKO.1-sh2-*SDHB*, and pLKO.1 scrambled shRNA plasmids were co-transfected with the delta 8.9 packaging plasmid and VSVG plasmid into Lenti-X™ 293 T cells using Lipofectamine 2000 (Invitrogen, Carlsbad, CA, USA). After overnight incubation, the cell culture medium was changed to DMEM supplemented with 30% FBS. The medium was harvested 48 h later and filtered through a 0.45-μM filter. The viral supernatant was used for infection of C1 and HEY cells with polybrene (4 μg/ml). Cells were infected overnight, and polyclonal populations of stable transductants were selected with puromycin (5 μg/ml) for 72 h.

### Western blot analysis

Cell lysates were prepared using RIPA lysis buffer (Sigma-Aldrich) containing a protease inhibitor cocktail (Roche, Basel, Switzerland). Protein concentrations were quantified by the BCA protein assay (Thermo Scientific, Rockford, IL, USA). Fifty micrograms of lysate was loaded onto a gradient gel (BioRad, Hercules, CA, USA) and subjected to gel electrophoresis. Semi-dry protein transfer to a PVDF nitrocellulose membrane (BioRad) was performed using the Transblot Turbo transfer system (BioRad). The membrane was then blocked in Odyssey blocking buffer (LiCOR, Lincoln, NE, USA) for 30 min and incubated with the appropriate antibody overnight at 4°C. Antibodies included E-cadherin (BD Biosciences, San Jose, CA, USA), β-actin (Abcam, Cambridge, MA, USA), Complex II Western Blot Antibody Cocktail that detects SDHB (30 kDa) and Complex Va (60 kDa) (Abcam, Burlungame, CA, USA), Methyl-Histone H3 Antibody Sampler Kit (Cell Signaling, Danvers, MA, USA), and SDHB (Santa Cruz Biotechnology, Dallas, TX, USA). Membranes were then washed with TBS-T, incubated with the appropriate secondary antibody for 1 h at room temperature and then washed with TBS-T. The membrane signal was subsequently analyzed by the LiCOR Odyssey system (LiCOR Biosciences, Frankfurt, Germany).

### qRT-PCR analysis

RNA was extracted using the RNeasy Miniprep Kit (Qiagen, Valencia, CA, USA) and was reverse transcribed to cDNA using the Quantitect Reverse Transcription Kit (Qiagen). For qRT-PCR, 50 ng of cDNA was mixed with the appropriate primers and the iQ SYBR-Green Supermix (BioRad) in a 96-well plate format. The qRT-PCR reaction was performed using the CFX96 Real-Time System (BioRad). Primer sequences are listed in Additional file 1: Table S1.

### Database mining

OncoPrints and the mRNA vs. CAN plot were generated using the cBio Cancer Genomics Portal (http://www.cbioportal.org) [22]. Tumorscape was analyzed as described [23].

### Immunofluorescence microscopy

Cells grown on coverslips were fixed with 3% paraformaldehyde for 20 min, washed with PBS, then permeabilized with 0.1% Triton X-100/PBS for 10 min. This was followed by incubation for 1 h at 37°C with E-cadherin antibody (1:1,000 dilution; BD Biosciences) or SDHB antibody (1:100 dilution; Santa Cruz Biotechnology). After washing with PBS, cells were incubated with appropriate fluorescent secondary antibody (1:1,000 dilution; Invitrogen) for 30 min at 37°C. The coverslips were mounted on slides with Vectashield mounting medium with DAPI (Vector Labs, Burlingame, CA, USA).

### Succinate dehydrogenase assay

The SDH assay was performed using the Succinate Dehydrogenase Activity Colorimetric Assay Kit (Biovision, Milpitas, California, USA). Briefly, 1 × 10^7^ cells were dounce homogenized in 1 ml SDH assay buffer. Five microliters of cell lysate were added to a mixture containing SDH assay buffer, SDH substrate mix, and SDH probe. Absorbance readings at 600 nm were taken every 3 min for a total of 30 min. SDH activity was determined by the formula *B*/(Δ*T* × *V*) × Dilution factor, where *B* = amount of reduced DCIP from standard curve (nmol), Δ*T* = reaction time (min), *V* = sample volume added into the reaction well (μl), and *D* = dilution factor.

### Cell proliferation and drug treatment

Cells were seeded onto 6-well or 96-well plates at 5 × 10^4^ or 1 × 10^3^ cells/well, respectively. For the glucose and glutamine withdrawal experiments, the medium was changed to DMEM + 10% FBS with indicated concentrations of glucose or glutamine 24 h post-seeding. To assay cell proliferation, triplicate cultures were trypsinized at the indicated time points and counted using the trypan blue exclusion method. To assay metformin sensitivity, various concentrations of metformin were added to cultures in a 6-well plate 24 h post-seeding and cells were stained with crystal violet at the indicated time points.

### Soft agar growth

To assay anchorage-independent growth, 1 × 10^4^ cells were mixed with 0.5% agarose and overlayed on a 0.9% agarose base layer in a 6-well plate. Plates were incubated at 37°C for 3 weeks in a 5% CO_2_ incubator. Colonies were stained with p-iodonitrotetrazolium chloride overnight and counted using a stereomicroscope. Colony size was measured using QCapture Pro™ (Q Imaging, Surrey, BC, Canada).

### Isotope labeling studies

For carbon tracing through central carbon metabolism, cells were cultured for approximately 24 h in 10-cm plates in glucose- and glutamine-free DMEM (Sigma D5030, Sigma-Aldrich) containing 10% dialyzed FBS, 1% streptomycin/penicillin, naturally labeled 4 mM glutamine or 25 mM glucose, and the appropriate tracer, [U-^13^C_6_]glucose or [U-^13^C_5_]glutamine (Cambridge Isotopes Laboratories, Inc., Cambridge, MA, USA). After incubation, the medium was aspirated and each plate was rinsed with 10 ml ice-cold saline. The saline was aspirated, and cells were quenched with 2.89 ml of −20°C HPLC-grade methanol. After adding 1.74 ml of ice water, the cells were scraped with a cell lifter and collected in 15-ml conical tubes. Chloroform (2.89 ml at −20°C) was added to each tube and vortexed for 10 min at 4°C. Extracts were centrifuged at 4,000 × *g* for 15 min at 4°C. The upper aqueous phase was collected in a separate tube and evaporated under nitrogen for polar metabolite analysis. The metabolites were analyzed using liquid chromatography tandem mass spectrometry (LC-MS/MS) using a variation of the method described previously [24]. A Paradigm MS4 HPLC (Michrom Bioresources, Auburn, CA, USA) and a Synergi Hydro column (4 μm particle size, 80 Å, 150 mm × 2 mm, from Phenomenex, Torrance, CA, USA) were used for the separation of metabolites by polarity. Prior to column separation, the samples were loaded onto a trapping column (C18, 4 mm × 2 mm, from Phenomenex) and washed for 30 s with HPLC grade water containing 10 mM tributylamine and 15 mM acetic acid for rapid desalting. HPLC separation was coupled with negative-mode electrospray ionization (ESI) to a TSQ Vantage triple stage quadrupole mass spectrometer (Thermo Scientific, Waltham, MA, USA) operating in multiple reactions monitoring (MRM) mode. The LC parameters were as follows: autosampler temperature, 10°C; injection volume, 10 μl; column temperature, room temperature; and flow rate, 200 μl/minute. The LC solvents were Solvent A: 10 mM tributylamine and 15 mM acetic acid in 97:3 water:methanol (pH 4.95), and Solvent B: methanol. Elution from the column was performed over 50 minutes with the following gradient: *t* = 0, 0% B; *t* = 5, 0% B; *t* = 10, 20% B; *t* = 20, 20% B; *t* = 35, 65% B; *t* = 38, 95% B; *t* = 42, 95% B, *t* = 43, 0% B; *t* = 50, 0% B. ESI spray voltage was 3,000 V. Nitrogen was used as sheath gas at 30 psi and as the auxiliary gas at 10 psi, and argon as the collision gas at 1.5 mTorr, with the capillary temperature at 325°C. Scan time for each MRM transition was 0.1 second with a scan width of 1 m/z. The LC runs were divided into time segments, with the MRM scans within each time segment containing compounds eluting during that time interval. For compounds eluting near boundaries between time segments, the MRM scan corresponding to the compound was conducted in both time segments. Instrument control, chromatographic control, and data acquisition were performed by the Xcalibar software (Thermo Scientific). Data analysis was performed using MAVEN [25, 26]. The protein content of extracted cells was determined from the dried protein fraction from each extraction, which was incubated overnight in 2 ml of 0.2 M KOH and quantified by Bradford assay. Isotope labeling data was corrected for the natural abundance of different isotopes using IsoCor [27].

### Glucose consumption and lactate excretion rates

Glucose and lactate levels in the media were analyzed at time 0 and after 72 h using LC-MS/MS. An H-Class UPLC system and AQCUITY UPLC BEH Amide column (2.1 × 100 mm, 1.7 μm particle size, from Waters) were used for the separation of glucose and lactate. UPLC separation was coupled with negative-mode ESI to a Waters Xevo TQ-S mass spectrometer operating in MRM mode. The LC parameters were as follows: autosampler temperature, 5°C; injection volume, 5 μl; column temperature, 50°C; and flow rate, 400 μl/min. The LC solvents were solvent A, 50 mM ammonium formate in water (pH 3) and solvent B, acetonitrile. Elution from the column was performed over 2 min with an isocratic gradient of 40% solvent A and 60% solvent B. The capillary voltage was 2.92 kV, and the cone voltage was 50 V. The flow rates of cone gas and desolvation gas were 150 and 600 L/h, respectively. The source temperature was 150°C, and the desolvation temperature was 500°C. Argon was used as collision gas at 1.5 mTorr. Collision energies and source cone potentials were optimized for each transition using Waters QuanOptimize software. Data were acquired using MassLynx 4.1 and QuanLynx software. The following equation was used to calculate metabolite consumption/excretion per 10^6^ cells per hour, denoted as *α*:


where


and *X* is the cell number and *t* is the time in hours.

### Cellular oxygen consumption and extracellular acidification rates

Cellular metabolic rates were measured using an XF24 Analyzer (Seahorse Bioscience, North Billerica, MA, USA) as previously described [28]. Mixing, waiting, and measurement times were 4, 2, and 2 min and 2, 3, and 3 min for C1 and HEY cells, respectively. The measurements were normalized against protein concentration. The concentrations for compounds injected during the analysis were 100 μM 2,4 dinitrophenol, 0.5 μM oligomycin, 1 μM FCCP, 1 μM rotenone-myxothiazol and 0.75 μM oligomycin, 0.5 μM FCCP, 0.75 μM rotenone-myxothiazol for C1 cells and HEY cells, respectively. Data was obtained using the XF24 Analyzer software.

### RNA-seq

Extracted RNA was enriched using the Ribominus™ Eukaryote Kit (Invitrogen). The Ion PI™ Template OT2 200 Kit (Invitrogen) was used to prepare the RNA library for sequencing. Sequencing was performed using the Ion Proton™ Sequencer (Life Technologies, Grand Island, NY, USA). Fragments per kilobase of exon per million reads (FPKM) were calculated using TopHAT and Cufflinks [29].

### Gene set analysis

Genes with FPKM smaller than 5 were discarded, leaving a total of 6,503 genes for further analysis. The log2 fold change (i.e., log2 of the FPKM ratio) was used as the gene-level statistic. The gene set analysis was carried out using the R/Bioconductor package *piano* [30]. Three types of gene set collections were used, describing the association between (i) genes and metabolites, (ii) genes and metabolic pathways, and (iii) Gene Ontology (GO) terms.

The human genome-scale metabolic network HMR2 was used to define the metabolite gene sets [31]. HMR2 contains gene associations for its enzymatic reactions, so for each metabolite, all genes that are associated with reactions in which the metabolite takes part are grouped into a gene set. The metabolic pathway gene sets were defined by all genes associated to reactions belonging to an individual pathway. Each set of human genes was translated into a set of mouse genes by mapping the human Ensembl gene identifiers to mouse Ensembl gene identifiers using the R/Bioconductor package *biomaRt* [32]. This package was also used to acquire the GO term gene. Gene set analysis was carried out separately for the metabolites, pathways, and GO terms. In all cases, the gene set statistics were calculated using *piano* with the median method and the gene set *p* values were calculated using gene permutation (10,000 permutations). The *piano* package calculates scores for several directionality classes. We primarily relied on the distinct-directional class for the identification of gene sets that are coordinately regulated in a distinct direction (up or down). Supplemental materials and methods are found in Additional file 2.

## Results

### SDH pathway members are downregulated in serous ovarian cancer

**Figure 1:**
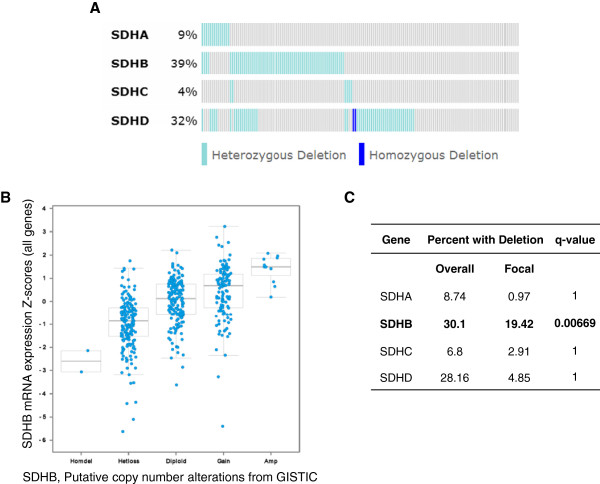
**SDH components are downregulated in serous ovarian cancer. (A)** Oncoprint depicting 575 patient samples with genetic loss of SDH subunits. Light blue and dark blue indicate heterozygous deletion and homozygous deletion, respectively. **(B)** A plot showing the relationship between *SDHB* mRNA abundance and copy number alteration of the *SDHB* gene. **(C)** Tumorscape copy number data for SDH subunits tabulated. Bold face indicates significance (*q* value < .01).

The Cancer Genome Atlas (TCGA) provides an exhaustive catalog of molecular changes for specific cancer types [4]. An analysis of the TCGA ovarian cancer dataset revealed significant genomic deletions of SDH members in 67% of serous ovarian cancer patient samples, with heterozygous deletion of *SDHB* found in 39% of the samples (Figure 1A). Heterozygous loss of *SDHB* correlated with decreased *SDHB* mRNA levels relative to diploid samples (Figure 1B). Statistically significant focal deletion of *SDHB* (*q* < .01) was confirmed in Tumorscape, a dataset that includes whole genome analyses of somatic copy number alterations (CNA) in 110 ovarian cancer samples [23] (Figure 1C). Recurrent hemizygous deletions have been found to enrich for genes involved in growth inhibition thereby optimizing proliferative potential [33, 34]. It has been proposed that the majority of tumor suppressor genes are likely to be haploinsufficient and selected for during the evolution of a cancer [34]. Homozygous focal deletions are rare in ovarian cancer [4], suggesting that haploinsufficiency may be the preferred mechanism for tumor progression. Immunohistochemical staining of ovarian carcinoma tissue microarray was used to determine levels of SDHB protein in malignant tumor cells relative to adjacent nonmalignant stroma. SDHB protein was detected in cytoplasmic granules in most cells, which is consistent with its predicted localization at the mitochondrial membrane. Of 211 ovarian cancer samples that had sufficient stromal and epithelial components for evaluation, seven samples exhibited significantly decreased levels of SDHB in tumor epithelia relative to tumor stroma (Additional file 3: Figure S1). Together, these data suggest that decreased SDH activity through altered expression of *SDHB* may be biologically relevant in a subset of ovarian cancers.

### Knockdown of *Sdhb*results in EMT in mouse ovarian cancer cells

**Figure 2:**
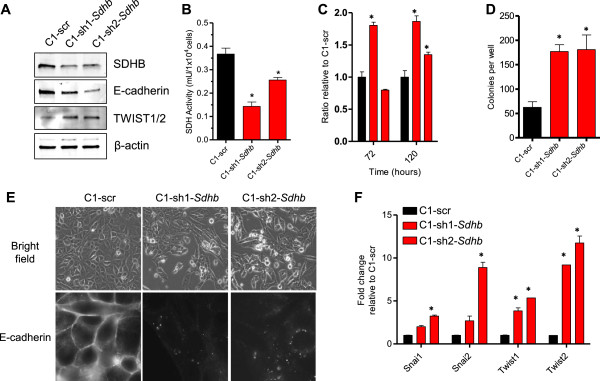
***Sdhb*** **knockdown in mouse ovarian cancer cells leads to EMT. (A)** Western blot detection of SDHB, E-cadherin, and TWIST2 after shRNA-mediated knockdown of *Sdhb*. β-actin was used as a loading control. **(B)** SDH assay illustrating the decrease in SDH activity upon knockdown of *Sdhb*. **(C)** Cell proliferation with counts taken at indicated time points. C1-scr is in black, while C1-sh1-*Sdhb* and C1-sh2-*Sdhb* are in red. Ratios are relative to C1-scr at the specified time point. **(D)** Formation of colonies in soft agar. **(E)** Bright field cell morphology and immunofluorescent visualization of E-cadherin. **(F)** Quantification of *Snai1*, *Snai2*, *Twist1*, and *Twist2* mRNA by qRT-PCR. Gapdh was used as a control. Statistical significance is based upon student *t*-test. Experiments were performed in triplicate and repeated at least three times. *p value < .01.

Given our analyses of the TCGA and Tumorscape datasets, we focused on the most frequently downregulated SDH subunit, *Sdhb*. We utilized C1 mouse ovarian cancer cells that are *p53* deficient and express *C-myc* and *K-ras*^*G12D*^. These genetically defined cells have been previously used as a system to elucidate the function of putative oncogenes in ovarian cancer [19–21]. Two shRNA constructs targeting *Sdhb* were used to generate stable *Sdhb* knockdown cell lines. Western blotting showed that both constructs (sh1-*Sdhb* and sh2-*Sdhb*) decreased the amount of SDHB expression relative to the scrambled control (Figure 2A). This was further confirmed by immunofluorescence (Additional file 4: Figure S2). *Sdhb* knockdown cell lines (C1-sh1-*Sdhb* and C1-sh2-*Sdhb*) showed a significant decrease in SDH activity relative to the control (Figure 2B). Knockdown of *Sdhb* resulted in an increased proliferation rate and an enhanced ability to form colonies in soft agar relative to the control C1-scr cells (Figure 2C,D). This effect on soft agar growth was also seen upon *SDHB* knockdown in the human ovarian cancer cell line, HEY (Additional file 5: Figure S3A,B). In addition to their augmented growth capability, *Sdhb* knockdown cells were more elongated and spindle-shaped compared to the cobblestone morphology of the control cells (Figure 2E). This morphological change resembled EMT. To determine the involvement of EMT at the molecular level, we analyzed *Sdhb* knockdown and control cells for membrane localization of E-cadherin, which is frequently lost during EMT. Immunofluorescence analysis revealed E-cadherin staining at the plasma membrane in C1-scr cells (Figure 2E). In contrast, E-cadherin localized within cytoplasmic punctate structures in *Sdhb* knockdown cells (Figure 2E). Quantitative RT-PCR analysis of mRNA levels of various transcription factors involved in EMT (*Snai1*, *Snai2*, *Twist1*, and *Twist2*) revealed their upregulation in *Sdhb* knockdown cells relative to the control cells, with the most dramatic increase in *Twist2* (Figure 2F). Decreased expression of E-cadherin and increased expression of TWIST1/2 in *Sdhb* knockdown cells were also seen on the protein level (Figure 2A). Typically under induction of EMT, cells continue to proliferate at the same level or arrest to promote invasion and migration [35]. However, the increased proliferation in C1 mouse ovarian cancer cells upon *Sdhb* knockdown can be explained by the overriding proliferative signal in these cells due to *c-myc* overexpression. Indeed, several studies have shown that various EMT-inducing factors, especially TWIST1/2, synergize with other oncogenic aberrations to enhance proliferation [36–38]. These data suggest that *Sdhb* knockdown transforms mouse ovarian cancer cells to a more aggressive and mesenchymal state.

### Knockdown of *Sdhb*leads to histone hypermethylation

**Figure 3:**
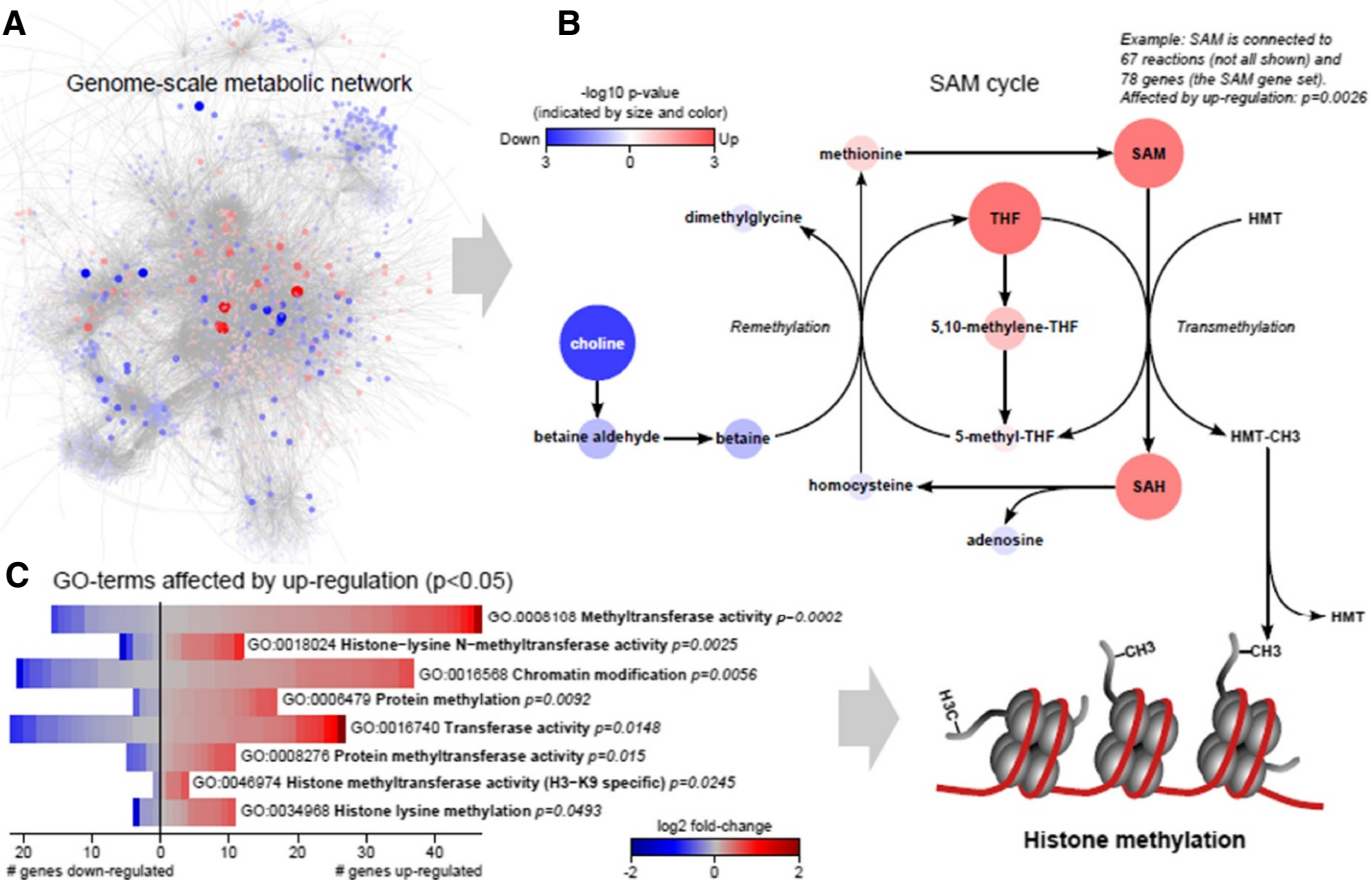
**Histone methylation is transcriptionally promoted after** ***Sdhb*** **knockdown. (A)** For topological analysis of metabolism, a human genome-scale metabolic network was used where the gene reaction association was mapped from human to mouse genes. Reporter metabolites, hotspots in metabolism around which significant transcriptional changes occur, are highlighted in red (upregulation) and blue (downregulation) nodes in the network when comparing C1-scr against C1-sh1-*Sdhb* cells. **(B)** A subnetwork found to be transcriptionally affected was S-Adenosyl methionine (SAM) metabolism. Both SAM, S-adenosyl homocysteine (SAH) and tetrahydrofolate (THF) were identified as reporter metabolites significantly (*p* < 0.005) affected by transcriptional upregulation of their neighboring enzymes. **(C)** A selection of GO terms related to histone methylation found significantly (*p* < 0.05) enriched by upregulated genes. The bar plot shows the number of upregulated and downregulated genes in each GO term and their individual fold changes in red and blue.

To identify how the loss of *Sdhb* led to EMT, we investigated the transcriptional regulation of cellular metabolism upon *Sdhb* knockdown. C1-scr and C1-sh1-*Sdhb* cells were first subjected to RNA-seq. The extracted data was then integrated with a recently published comprehensive human genome-scale metabolic network, HMR2 [39]. HMR2 represents a holistic view of metabolism by providing associations between metabolites, reactions, and their corresponding enzyme-coding genes, which we mapped to mouse homologs to match the RNA-seq gene annotation. For each metabolite, we constructed a gene set consisting of all enzymes catalyzing reactions in which the metabolite takes part, i.e., genes that control the flux through that metabolite. Next, we performed gene set analysis on the metabolite gene sets using the log2 fold changes of the genes to score each metabolite [30]. Gene sets (metabolites) found significant in this analysis are termed reporter metabolites [40] and represent hotspots in the metabolic network around which significant transcriptional regulation occurs (Figure 3A, Additional file 6: Table S2). Interestingly, S-adenosyl methionine (SAM), S-adenosyl homocysteine (SAH), and tetrahydrofolate (THF), relevant metabolites in the SAM cycle, were all identified as reporter metabolites (*p* < 0.005) influenced by transcriptional upregulation (Figure 3B). In the SAM cycle, the reactive methyl group in SAM is transferred to a substrate in a transmethylation reaction producing SAH. If the substrate is a histone methyltransferase (HMT), this can subsequently methylate the histones, thus influencing the epigenome.

To further validate the transcriptional influence on histone methylation, we performed an additional gene set analysis using GO terms as gene sets. In Figure 3C, GO terms significantly enriched with upregulated genes (*p* < 0.05) and relevant to histone methylation are shown, clearly indicating a pattern of increased histone methylation regulated on the transcriptional level (complete results are presented in Additional file 6: Table S2). In summary, *Sdhb* knockdown appears to be orchestrating a transcriptional program in which histone methylation is promoted or maintained, possibly through upregulation of the SAM cycle.

**Figure 4:**
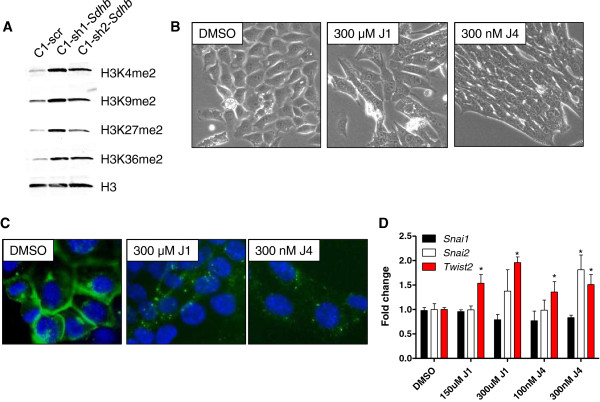
***Sdhb*** **knockdown leads to a hypermethylated epigenome; small-molecule inhibition of H3K27 demethylases phenocopies loss of** ***Sdhb.*** **(A)** Western blot of various histone methylation marks with Histone H3 as a loading control. **(B)** Bright field microscopy of untreated cells or cells treated with indicated concentrations of GSK-J1 or GSK-J4 for 4 days. **(C)** Immunofluorescent microscopy of cells stained for E-cadherin (green) and DAPI (blue). **(D)** Quantification of *Snai1*, *Snai2*, and *Twist2* mRNA relative to *Gapdh*. *p value < .01.

To confirm whether histone methylation was affected, we analyzed by Western blot various methylated histone marks in *Sdhb* knockdown and control cells. Indeed, *Sdhb* knockdown cells had elevated levels of histone H3 dimethylated K4, K9, K27, and K36 marks in comparison to control cells (Figure 4A). We further investigated the aberration in H3K27 methylation because of its known association with malignant progression [41]. There are two known H3K27 demethylases, JMJD3 and UTX, in mammalian cells. Highly selective H3K27 demethylase inhibitors, GSK-J1 and GSK-J4 (a more cell permeable derivative of GSK-J1), have been developed [42]. To determine whether chemical inhibition of H3K27 histone demethylases could lead to EMT, C1-scr cells were treated with DMSO or each inhibitor for 5 days. DMSO-treated cells exhibited cobblestone morphology, while treatment with GSK-J1 or GSK-J4 resulted in a spindle-shaped and elongated cell morphology analogous to *Sdhb* knockdown (Figure 4B). To confirm that EMT occurred in cells treated with H3K27 demethylase inhibitors, E-cadherin localization was analyzed by immunofluorescence. Indeed, E-cadherin was localized to punctate cytoplasmic structures in cells treated with GSK-J1 or GSK-J4 as opposed to the plasma membrane staining found in the DMSO-treated control cells (Figure 4C). Cells treated with GSK-J1 and GSK-J4 also had elevated levels of *Snai2* and *Twist2* mRNA relative to the DMSO-treated control cells as determined by qRT-PCR (Figure 4D). These data show that hypermethylation of H3K27 is sufficient to induce EMT, suggesting that the hypermethylated epigenome induced by the loss of *Sdhb* may be responsible for the EMT phenotype.

### *Sdhb*knockdown leads to reprogrammed glucose and glutamine utilization

**Figure 5:**
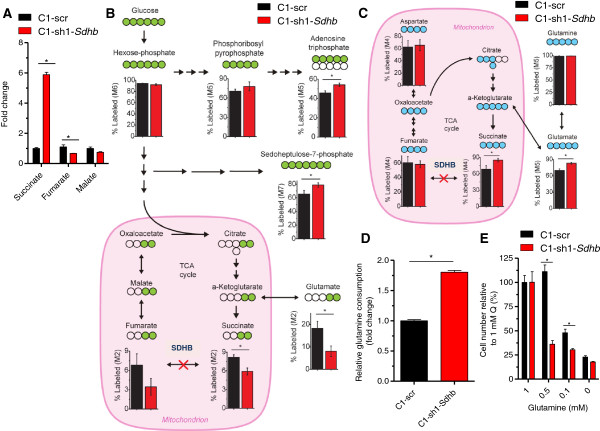
**Carbon source utilization is altered upon** ***Sdhb*** **knockdown. (A)** Mass spectrometry analysis of intracellular succinate, fumarate, and malate levels in *Sdhb* knockdown and scr control cells. Labeling of intracellular metabolites with **(B)** [U-^13^C]glucose or **(C)** [U-^13^C]glutamine was measured using mass spectrometry in *Sdhb* knockdown and scr control cells. Percent labeling of indicated isotopomers is shown. **(D)** Mass spectrometry analysis of extracellular glutamine in spent media of C1-scr and C1-sh1-*Sdhb* cells. Media was collected at 0 and 72 h. The *y*-axis depicts fold change of glutamine consumption relative to C1-scr cells. **(E)** Cells grown in normal media (1 mM glutamine) were switched to media with indicated concentrations of glutamine after 24 h. Cells were counted by trypan blue exclusion after 48 h. *p-value < .01.

Given the fact that SDH is intricately involved in central carbon metabolism through its role in the TCA cycle and ETC, we examined global metabolite levels in the C1-scr and C1-sh1-*Sdhb* cells by LC-MS/MS. As expected, *Sdhb* knockdown cells had elevated levels of succinate and decreased levels of fumarate and malate relative to C1-scr cells (Figure 5A). This metabolic signature confirms the decrease in SDH activity upon *Sdhb* knockdown. In addition to altering TCA cycle intermediates succinate, fumarate, and malate, *Sdhb* knockdown affected many intracellular metabolites including amino acids, nucleotides, and intermediates in glycolysis as well as the pentose phosphate pathway (Additional file 7: Figure S4). Interestingly, *Sdhb* knockdown led to a significant increase of methylmalonic acid, possibly due to the feedback inhibition of succinate-CoA ligase (SUCL) by succinate (Additional file 7: Figure S4).

To further probe metabolic alterations caused by *Sdhb* knockdown, we used isotope-labeled nutrients combined with mass spectrometry-based techniques to trace glucose and glutamine through central carbon metabolism. Analysis of intracellular metabolites after culturing cells in the presence of uniformly labeled ^13^C-glucose ([U-^13^C_6_]glucose) revealed that *Sdhb* knockdown increased glucose contribution to sedoheptulose-7-phosphate and adenosine triphosphate (ATP); in contrast, glucose contribution to TCA cycle intermediate succinate was decreased (Figure 5B). Consistently, succinate and glutamate labeling from uniformly labeled ^13^C-glutamine ([U-^13^C_5_]glutamine) was increased (Figure 5C). This increased glutamine utilization by *Sdhb* knockdown cells is highlighted by their increased uptake of glutamine relative to the control cells (Figure 5D). These data suggest that *Sdhb* knockdown increases glucose contribution to the pentose phosphate pathway and nucleotide biosynthesis to support rapid proliferation. Increased nucleotide synthesis from glucose is achieved by decreasing glucose contribution to the TCA cycle, which is compensated by increasing glutamine contribution to the TCA cycle. Intracellular pool levels also support a model in which nucleotide biosynthesis is upregulated, as many glycolytic and pentose phosphate pathway intermediates are increased in response to *Sdhb* knockdown (Additional file 7: Figure S4).

Given the increased use of glutamine in *Sdhb* knockdown cells, we hypothesized that these cells have an augmented dependency on glutamine for survival relative to the control cells. To test this, we assayed the effect of varying glutamine concentration on cell proliferation. Indeed, in lower glutamine concentrations, C1-sh1-*Sdhb* cells grew slower in comparison to the control C1-scr cells (Figure 5E). Therefore, knockdown of *Sdhb* leads to a greater reliance on glutamine for cell survival.

### *Sdhb*knockdown results in a mitochondrial reserve capacity defect that can be exploited by metformin

**Figure 6:**
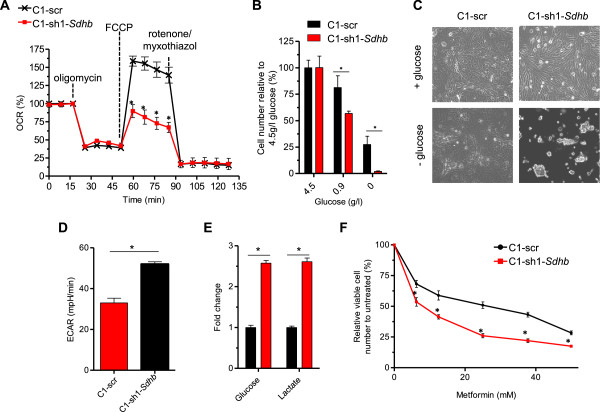
**The bioenergetics defect of** ***Sdhb*** **knockdown cells can be exploited with metformin. (A)** The oxygen consumption rate (OCR) was analyzed by the Seahorse XF24 Bioanalyzer in basal conditions and in response to sequential treatment with oligomycin (ATP synthase inhibitor), FCCP (dissipates mitochondrial membrane potential), and rotenone-myxothiazol (complex I inhibitor). **(B, C)** Cells grown in normal media were switched to media with or without glucose after 24 h. **(B)** After 48 h, cells were counted by trypan blue exclusion or **(C)** visualized by light microscopy. **(D)** The extracellular acidification rate (ECAR), an indicator of glycolysis, was analyzed by the Seahorse XF24 Bioanalyzer in basal conditions. **(E)** Mass spectrometry analysis of lactate and glucose in spent media of C1-scr and C1-sh1-*Sdhb* cells. Media was collected at 0 and 72 h. The *y*-axis depicts fold change of lactate secretion and glucose depletion relative to C1-scr cells. **(F)** Cells were treated with various concentrations of metformin for 3 days. Cells were visualized by crystal violet staining and extracted dye was quantified by a fluorescent plate reader.

Since *Sdhb* knockdown alters central carbon metabolism including the TCA cycle occurring in the mitochondria, we investigated the effect of *Sdhb* knockdown on mitochondrial activity. A key indicator of mitochondrial function is the oxygen consumption rate (OCR). To ascertain specific mitochondrial perturbations, the OCR of C1-scr and C1-sh1-*Sdhb* cells was determined during sequential treatment with compounds that modulate mitochondrial activity. Oligomycin, a F_1_F_0_ ATP synthase inhibitor, blocks proton movement, thereby inhibiting oxidative phosphorylation (OXPHOS). FCCP reestablishes proton movement by dissipating the mitochondrial membrane potential to stimulate maximal O_2_ consumption and electron transport [43]. Finally, rotenone/myxothiazol, complex I and III inhibitors, completely block mitochondrial respiration. Both cell lines had similar basal OCR and responses to oligomycin and rotenone (Figure 6A). However, the cell lines responded differently to FCCP treatment (Figure 6A). The calculated difference between FCCP-induced and basal OCR indicates reserve respiratory capacity. FCCP injection increased respiration 59% in C1-scr while spare respiratory capacity was nonexistent in C1-sh1-*Sdhb* cells (Figure 6A). Thus, C1-sh1-*Sdhb* cells respirate at their maximum capacity. *SDHB* knockdown in HEY cells resulted in a lower basal respiration implying a greater reliance on SDH respiration in this particular cell line (Additional file 5: Figure S3C). Therefore, knockdown of *Sdhb* decreases mitochondrial reserve respiratory capacity.

The reserve respiratory capacity must be sufficient to provide enough ATP during times of energy stress. If ATP demands are not met, affected cells risk being driven into senescence or cell death [44]. To determine whether the decreased reserve capacity of *Sdhb* knockdown cells rendered them sensitive to energy stress, C1-scr and C1-sh1-*Sdhb* cells were cultured in normal media then switched to media with varying amounts of glucose. Despite the previous observation that *Sdhb* knockdown cells grew at a faster rate than control cells under normal growth conditions (Figure 2B), C1-sh1-*Sdhb* cells grew at a slower rate than C1-scr cells upon glucose withdrawal (Figure 6B). In fact, the majority of Sdhb knockdown cells appeared to undergo apoptosis (Figure 6C). Consistent with the increased dependence of C1-sh1-*Sdhb* cells on glucose relative to C1-scr cells, C1-sh1-*Sdhb* cells were more glycolytic (Figure 6D), took up glucose and excreted lactate at increased levels relative to C1-scr cells (Figure 6E). We suspect that the effect of glucose starvation on the *Sdhb* knockdown cells is twofold, a failure to adapt to the decrease in glycolytic ATP and its reliance on the pentose phosphate pathway for nucleotide biosynthesis.

Since C1-sh1-*Sdhb* cells are hypersensitive to glucose starvation, we were interested if this bioenergetics deficiency could be therapeutically exploited. Metformin, a commonly used antidiabetic drug, inhibits Complex I in the ETC [45]. This reduces the cellular energy charge, a measure of the energetic state of the cell’s adenine nucleotide pool defined as ([ATP] + 0.5[ADP]) / ([ATP] + [ADP] + [AMP]) [46]. To determine if metformin-induced energy stress had a differential effect on C1-scr and C1-sh1-*Sdhb* cells, cells were exposed to various concentrations of metformin. C1-sh1-*Sdhb* cells were hypersensitive to metformin treatment relative to C1-scr cells (Figure 6F). Specifically, metformin inhibited growth of the control and *Sdhb* knockdown cells with IC50 values of 29.73 and 14.73 mM, respectively (Figure 6F). Taken together, these results implicate a crucial role for Sdhb in the response to bioenergetic insult.

## Discussion

Various mechanisms have been proposed for SDH-associated malignancy. Due to SDH’s role in the ETC, it has been suggested that reactive oxygen species (ROS) can be induced by SDH-inactivating mutations, which contributes to genomic instability, DNA damage, and other tumorigenic phenomena [47]. However, *Sdhb* knockdown did not lead to altered ROS levels, and the antioxidant N-acetylcysteine (NAC) had no effect on various Sdhb knockdown phenotypes in our system (Additional file 8: Figure S5A). Interestingly, many studies have focused on the idea that succinate itself can act as an oncometabolite. Due to its structural similarity to α-ketoglutarate (α-KG), succinate has been found to competitively inhibit α-KG-dependent dioxygenases. This phenomenon was first described linking succinate to HIF1α stabilization; succinate was found to directly inhibit prolyl hydroxylases which serve to degrade HIF1α [48, 49]. However, we did not find altered HIF signaling between the control and *Sdhb* knockdown cells (Additional file 8: Figure S5B). This may be due in part to the modestly elevated levels of succinate in our *Sdhb* knockdown cells, approximately six-fold vs. control (Figure 5A), in comparison to previous studies (approximately 100-fold in tumors and approximately 300-fold upon complete knockout of Sdhb in mouse cells) [16, 50]. It is also possible that HIF signaling by *SDHx* mutations is tissue-dependent, thus explaining the specific range of cancers induced by SDH inactivation. Recently, the repertoire of α-KG-dependent dioxygenases targeted by succinate has expanded to include epigenome-modifying enzymes such as the TET family of 5-methylcytosine hydroxylases and histone demethylases [51]. Inhibition of these enzymes by elevated levels of succinate leads to genome-wide histone and DNA methylation [51, 52]. In support of this idea, *SDHx-*related PGL tumors were found to display a hypermethylator phenotype, where epigenetic silencing was particularly severe in *SDHB*-mutated tumors [16]. This association between *SDHx* mutations and hypermethylation was also found in GIST [53]. Our data further support this model in which SDH inhibition leads to a hypermethylated epigenome. It has been suggested that a stronger hypermethylator phenotype, which associates with stronger downregulation of target genes, may contribute to the metastatic phenotype [16]. Our results are in line with this idea as aberrant hypermethylation of H3K27 in mouse ovarian cancer cells was sufficient for EMT, thereby implicating the link between epigenetic remodeling and cell state [16]. However, overexpression of the H3K27 demethylases, UTX and JMJD3, in *Sdhb* knockdown cells failed to reverse EMT, thereby indicating a higher complexity to the maintenance of the mesenchymal state (Additional file 9: Figure S6). This is likely due to transcriptional upregulation of metabolic and epigenetic networks to support this hypermethylated state. Therefore, SDH dysfunction is a contributor of the tumorigenic process via mitochondrion-to-nuclear signaling.

In addition to SDH activity being linked to epigenetic reprogramming, we also found that *Sdhb* knockdown leads to reprogrammed glucose and glutamine utilization. Specifically, increased glucose flux into the pentose phosphate pathway and increased glutamine usage in the TCA cycle. Interestingly, these phenomena mimic disruption of another TCA cycle enzyme, fumarate hydratase (FH), in mouse kidney cells [54]. The mechanism by which SDH dysfunction leads to these effects is unknown. One possibility is that the global epigenetic changes induced by *Sdhb* knockdown may lead to an altered transcriptome as evidenced by pathway enrichment analysis of the RNA-seq data (Additional file 7: Figure S4). The analysis indicated that the pentose phosphate pathway and nucleotide metabolism gene sets were both upregulated. The gene set analysis also showed that genes associated with oxidative phosphorylation were significantly downregulated (Additional file 10: Figure S7). In similar fashion, the *MYC* proto-oncogene has been shown to directly control the transcription of various genes involved in several metabolic pathways including glycolysis, the pentose phosphate pathway, and glutaminolysis [55, 56]. Also, the glycolytic enzyme fructose-1,6-biphosphatase (FBP1) has been shown to influence metabolic reprogramming to impact EMT progression [57]. How SDH dysfunction affects the regulation of these enzymes will be an interesting subject of future research. Interestingly, an association between glutamine dependence and cancer invasiveness has been recently found in ovarian cancer, thereby providing further support for carbon source reprogramming to support different ovarian cancer states [58].

## Conclusions

Although SDH dysfunction leads to enhanced cancer aggressiveness, it also results in metabolic vulnerabilities due to its involvement in both the TCA cycle and ETC. We showed that altered carbon source utilization in *Sdhb* knockdown cells renders them hypersensitive to both glucose and glutamine starvation. Also, by analyzing mitochondrial function, we found that SDH dysfunction leads to a decreased mitochondrial reserve capacity that can be exploited by energy stress caused by either glucose withdrawal or metformin treatment. Interestingly, it has been shown that combinatorial treatment with metformin and the glycolytic inhibitor 2-deoxyglucose are effective in a broad spectrum of pre-clinical cancer models [59]. Therefore, it is worth exploring the possibility that tumors with *SDHx* mutations can be therapeutically targeted by exploiting their metabolic state.

## Electronic supplementary material

Additional file 1: Table S1: shRNA and qRT-PCR sequences. (XLSX 12 KB)

Additional file 2: **Supplementary materials and methods.** (DOCX 20 KB)

Additional file 3: Figure S1: SDHB protein expression in high-grade ovarian carcinoma. Representative images of tumors with **(A)** ubiquitous SDHB expression in epithelial and stromal cells and **(B)** decreased SDHB expression in epithelial cells relative to stromal cells. T, tumor; S, stroma. (PDF 362 KB)

Additional file 4: Figure S2: Immunofluorescence staining of SDHB in C1-scr and *Sdhb* knockdown cells. (PDF 361 KB)

Additional file 5: Figure S3: *SDHB* knockdown in HEY cells results in enhanced anchorage-independent growth and a defect in mitochondrial function. **(A)** Western blot detection of SDHB after shRNA-mediated knockdown. Complex V alpha was used as a loading control. **(B)** Colony size in soft agar. **(C)** OCR was analyzed by the Seahorse XF24 Bioanalyzer in basal conditions and in response to sequential treatment with oligomycin, FCCP, and rotenone-myxothiazol. *p value < .01. (PDF 99 KB)

Additional file 6: Table S2: Table of reporter metabolites and GO terms. (XLSX 613 KB)

Additional file 7: Figure S4: Global analysis of metabolites in C1-scr and C1-sh1-*Sdhb* cells. Steady state levels of metabolites in control and *Sdhb* knockdown cells were determined by mass spectrometry. A heat map was generated using Cluster 3.0 for unsupervised clustering and JavaTreeView for visualization. (PDF 363 KB)

Additional file 8: Figure S5: *Sdhb* knockdown does not affect ROS levels or HIF signaling in C1 cells. **(A)** C1-scr and C1-sh1-*Sdhb* cells were incubated with MitoTracker® Red CM-H2XRos to determine relative ROS levels. No significant difference in probe intensity was observed. **(B)** HIF-response element luciferase activity in *Sdhb* knockdown and control cells. Data are normalized to renilla luciferase activity and shown relative to C1-scr cells. (PDF 107 KB)

Additional file 9: Figure S6: Overexpression of H3K27 demethylases, JMJD3 and UTX, does not promote MET in *Sdhb* knockdown cells. C1-sh1-*Sdhb* and C1-sh2-*Sdhb* cells were transfected with HA-tagged JMJD3, UTX, or GFP constructs. Lysates were collected 7 days post-transfection. Western blot detection of HA-tagged JMJD3 and UTX, E-cadherin, and β-actin. (PDF 67 KB)

Additional file 10: Figure S7: *Sdhb* knockdown affects the transcriptional regulation of the metabolism of amino acids, nucleotides, and oxidative phosphorylation. In order to identify metabolic pathways influenced by transcriptional regulation, we performed a gene set analysis using the pathways defined by the human genome-scale metabolic network HMR2. The resulting heat map shows the top significant pathways affected by transcriptional changes. The leftmost and rightmost columns show *p* values for the pathways being coordinately regulated in a distinct direction. The second columns from the left and right show the *p* values for pathways having a significant subset of upregulated or downregulated genes. The middle column shows the *p* values for pathways affected by transcriptional regulation in general, regardless of the direction. The pentose phosphate pathway, providing backbones for both nucleotides and amino acids, is upregulated. This pattern is also seen for pathways related to nucleotide metabolism and amino acid metabolism (marked in red). Oxidative phosphorylation is significantly downregulated (marked in blue). For the relevant pathways, *p* values <0.04 are marked with a black box. (PDF 100 KB)

## Abbreviations

SDH: succinate dehydrogenase
EMT: epithelial-to-mesenchymal transition
TCA: the citric acid cycle
ETC.: electron transport chain
SDHAF1/SDHAF2: succinate dehydrogenase assembly factor
PCC: hereditary pheochromocytoma
PGL: paraganglioma
GIST: gastrointestinal stromal tumor
FCCP: carbonyl cyanide 4-(trifluoromethoxy)phenylhydrazone
TCGA: the Cancer Genome Atlas
CNA: copy number alterations
HMR: human genome-scale metabolic network
SAM: S-adenosyl methionine
SAH: S-adenosyl homocysteine
THF: tetrahydrofolate
OCR: oxygen consumption rate
OXPHOS: oxidative phosphorylation
ECAR: extracellular acidification rate
ROS: reactive oxygen species
NAC: N-acetylcysteine
FH: fumarate hydratase.
