# Supplementary material for: Succinate dehydrogenase inhibition leads to epithelial-mesenchymal transition and reprogrammed carbon metabolism
Source: Cancer Metab. 2014 Dec 15;2:21. doi: 10.1186/2049-3002-2-21 (PMC4322794; doi:10.1186/2049-3002-2-21)
Supplement: Supplementary file 2 — Additional file 2: Supplementary materials and methods. (DOCX 20 KB) [file 40170_2014_142_MOESM2_ESM.docx]

**Supplementary Materials and Methods**

**Plasmids and Reagents**

MitoTracker® Red CM-H2XRos was purchased from Life Technologies. The HIF-response element (HRE)-luciferase plasmid was obtained from Affymetrix (Santa Clara, CA, USA). The pRenillaLuciferase(RL)-null vector was obtained from Promega (Madison, WI, USA).

**Immunohistochemical detection of SDHB in ovarian cancer**

A tissue microarray representing duplicate or triplicate cores from serous ovarian carcinomas on four slides was immunostained with SDHB-specific antibody (SigmaAldrich, 1:500), which has been tested by the Human Protein Atlas (http://www.proteinatlas.org/). Cores containing normal liver tissue were used as a positive control for immunodetection. Immunostaining was performed as previously described [[1](#_ENREF_1)]. To determine if SDHB expression is altered in cancer, the intensity of staining in epithelial cancer cells was compared to the intensity of staining in adjacent stromal cells.

**Reactive oxygen species (ROS) detection**

Cells were stained with 100 nM MitoTracker® Red CM-H2XRos in DMEM for 30 minutes at 37°C in a CO_2_ incubator. Cells were then washed with DMEM and examined for mitochondrial ROS staining.

**HIF1 luciferase assay**

Cells were transfected with the HRE-luc and RL-null vector using Lipofectamine 2000 (Invitrogen). Dual-luciferase assay (Promega) was performed on cells after 48 hours per the manufacturer’s protocol.

**Overexpression of H3K27 demethylases**

Sdhb knockdown cells were transfected with pCMV-HA-JMJD3 (Addgene, Plasmid 24167) and pCMV-HA-UTX (Addgene, Plasmid 24168)[[2](#_ENREF_2)] using Lipofectamine 2000 (Invitrogen). Cell lysates were collected in RIPA buffer at two time points, 3 and 7 days.

1. Miao J, Mu D, Ergel B, Singavarapu R, Duan Z, Powers S, Oliva E, Orsulic S: **Hepsin colocalizes with desmosomes and induces progression of ovarian cancer in a mouse model.** *Int J Cancer* 2008, **123:**2041-2047.

2. Agger K, Cloos PA, Christensen J, Pasini D, Rose S, Rappsilber J, Issaeva I, Canaani E, Salcini AE, Helin K: **UTX and JMJD3 are histone H3K27 demethylases involved in HOX gene regulation and development.** *Nature* 2007, **449:**731-734.
