# Supplementary figures and images for: Succinate dehydrogenase inhibition leads to epithelial-mesenchymal transition and reprogrammed carbon metabolism
Source: Cancer Metab. 2014 Dec 15;2:21. doi: 10.1186/2049-3002-2-21 (PMC4322794; doi:10.1186/2049-3002-2-21)

**A**

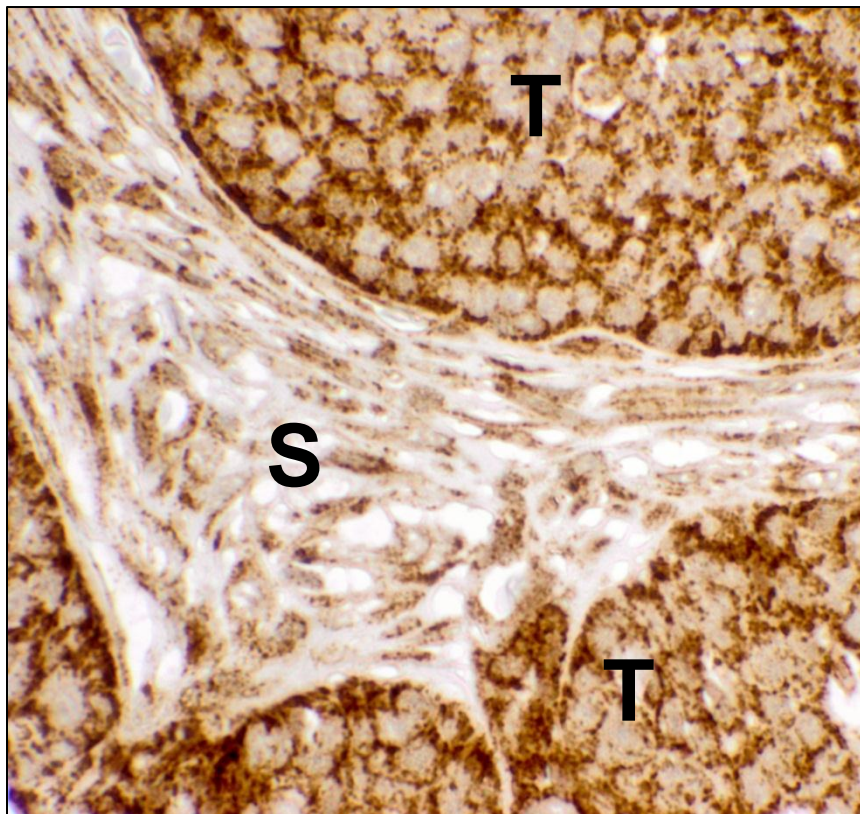

**B**

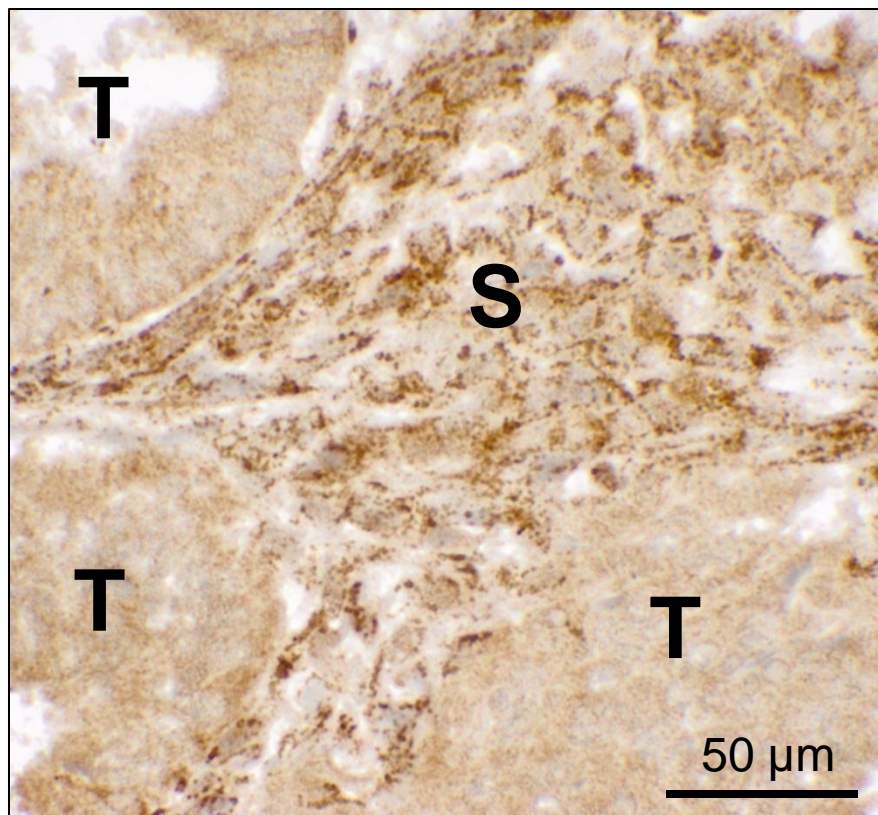

Supplement: Supplementary file 3 — Additional file 3: Figure S1: SDHB protein expression in high-grade ovarian carcinoma. Representative images of tumors with (A) ubiquitous SDHB expression in epithelial and stromal cells and (B) decreased SDHB expression in epithelial cells relative to stromal cells. T, tumor; S, stroma. (PDF 362 KB) [file 40170_2014_142_MOESM3_ESM.pdf]

$\alpha$ SDHB

Bright field

Composite

C1-scr

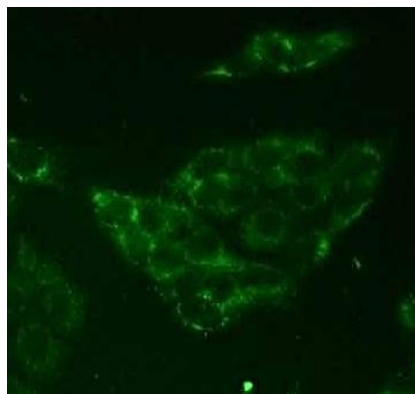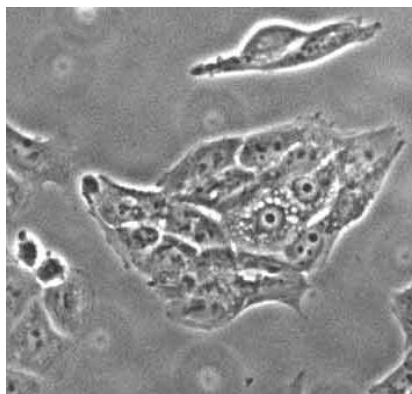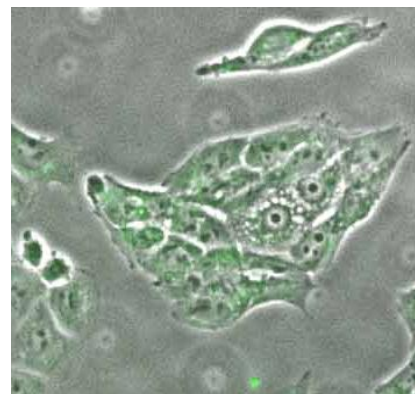

C1-sh1-*Sdhb*

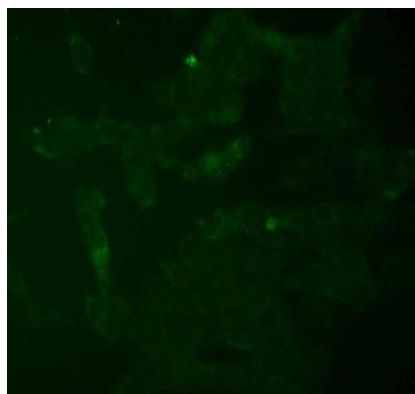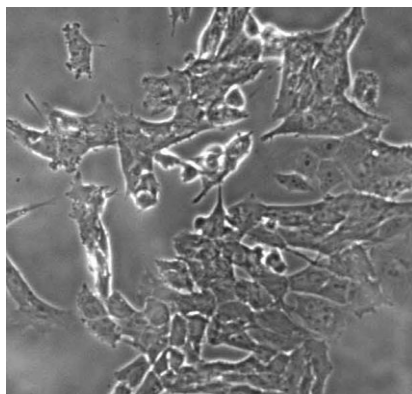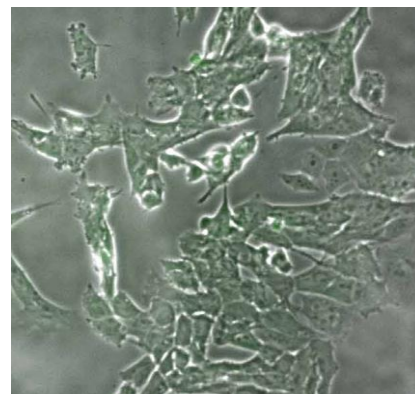

C1-sh2-*Sdhb*

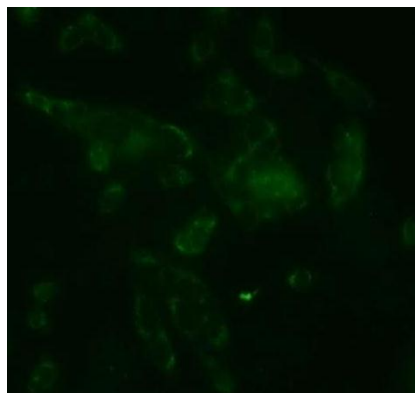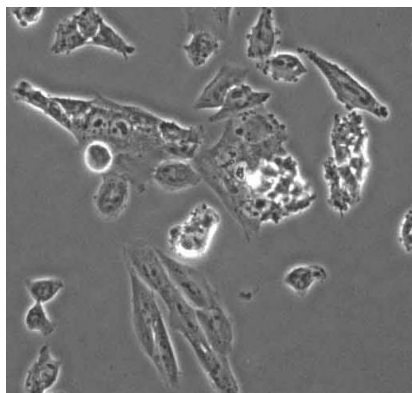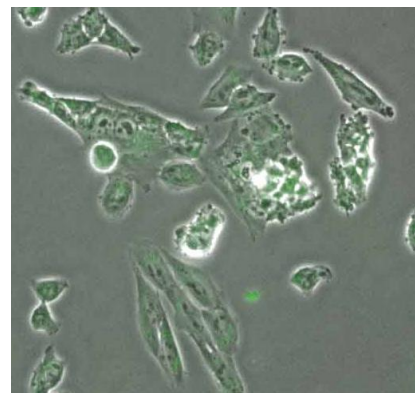

Supplement: Supplementary file 4 — Additional file 4: Figure S2: Immunofluorescence staining of SDHB in C1-scr and Sdhb knockdown cells. (PDF 361 KB) [file 40170_2014_142_MOESM4_ESM.pdf]

**A**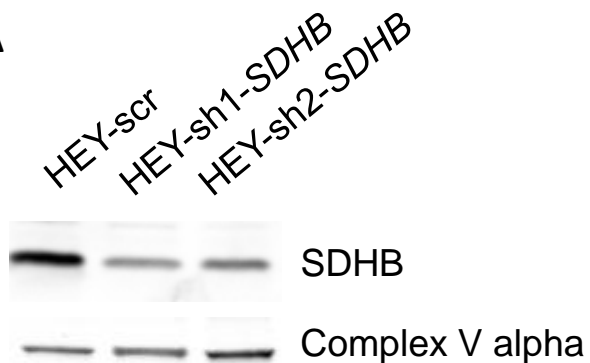**B**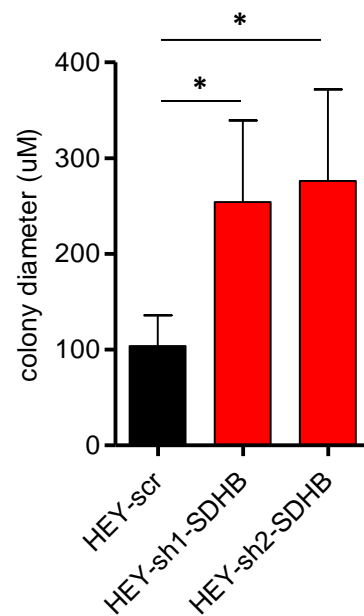**C**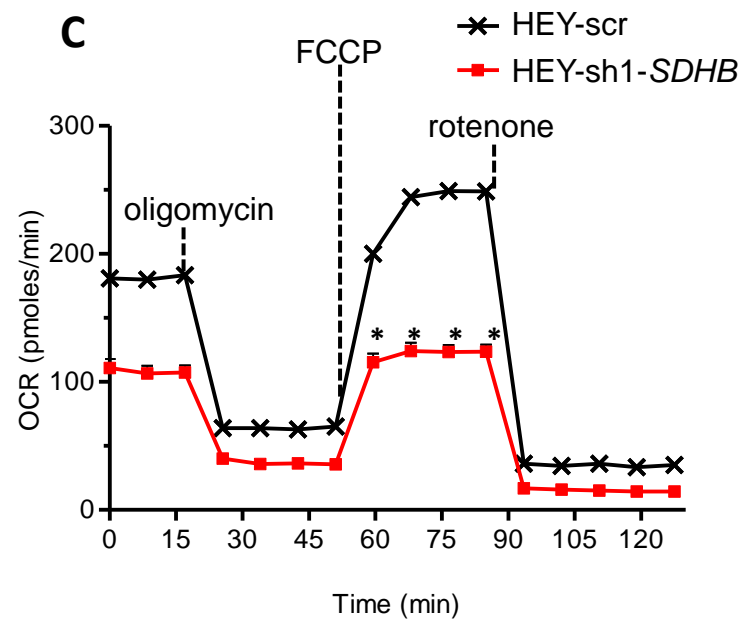

Supplement: Supplementary file 5 — Additional file 5: Figure S3: SDHB knockdown in HEY cells results in enhanced anchorage-independent growth and a defect in mitochondrial function. (A) Western blot detection of SDHB after shRNA-mediated knockdown. Complex V alpha was used as a loading control. (B) Colony size in soft agar. (C) OCR was analyzed by the Seahorse XF24 Bioanalyzer in basal conditions and in response to sequential treatment with oligomycin, FCCP, and rotenone-myxothiazol. *p value < .01. (PDF 99 KB) [file 40170_2014_142_MOESM5_ESM.pdf]

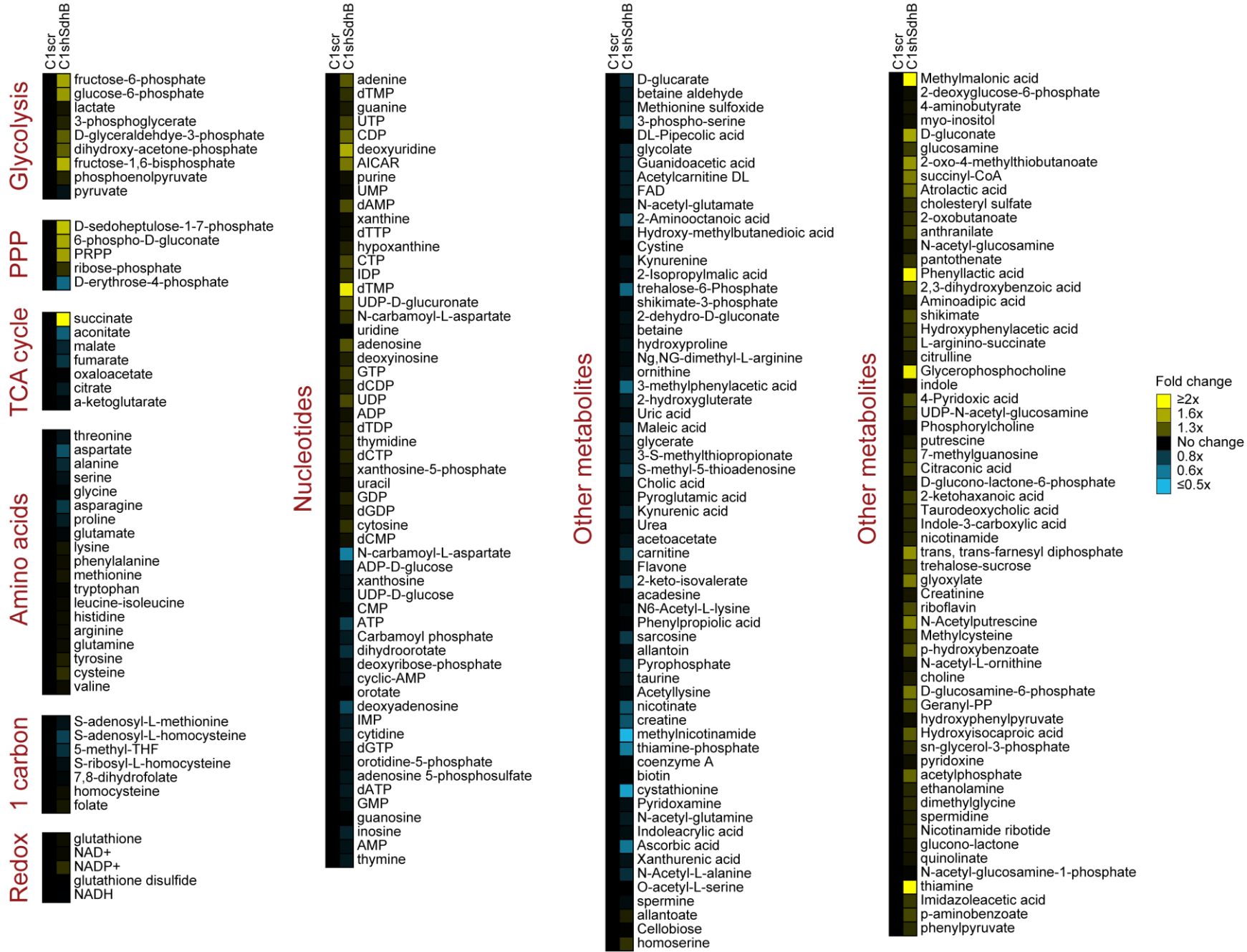

Supplement: Supplementary file 7 — Additional file 7: Figure S4: Global analysis of metabolites in C1-scr and C1-sh1-Sdhb cells. Steady state levels of metabolites in control and Sdhb knockdown cells were determined by mass spectrometry. A heat map was generated using Cluster 3.0 for unsupervised clustering and JavaTreeView for visualization. (PDF 363 KB) [file 40170_2014_142_MOESM7_ESM.pdf]

**A**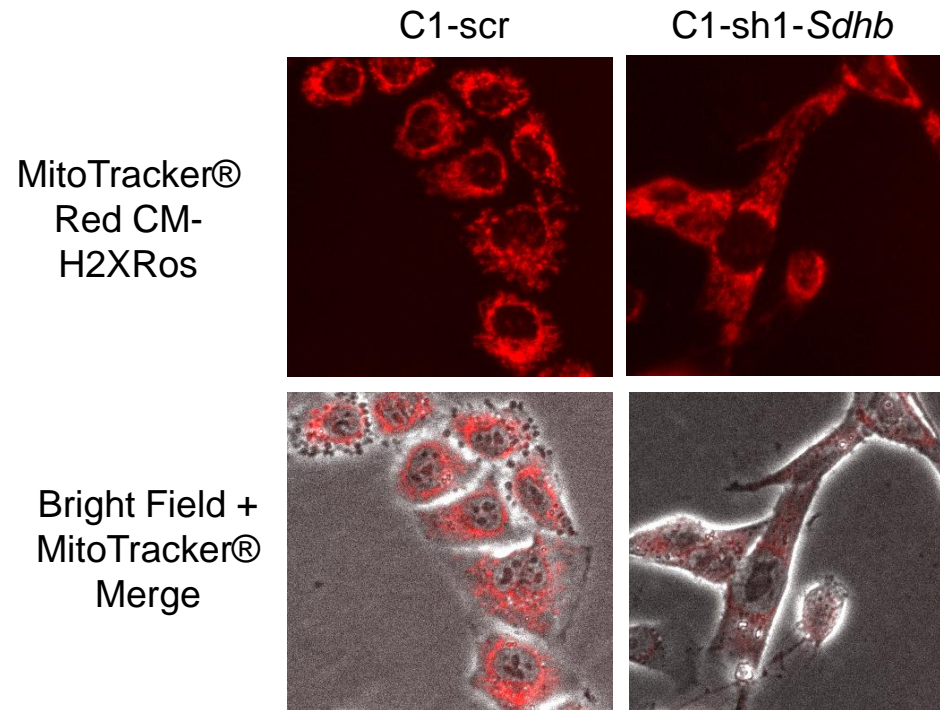**B**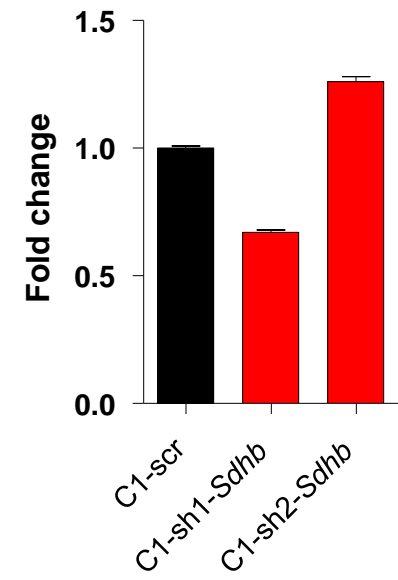

Supplement: Supplementary file 8 — Additional file 8: Figure S5: Sdhb knockdown does not affect ROS levels or HIF signaling in C1 cells. (A) C1-scr and C1-sh1-Sdhb cells were incubated with MitoTracker® Red CM-H2XRos to determine relative ROS levels. No significant difference in probe intensity was observed. (B) HIF-response element luciferase activity in Sdhb knockdown and control cells. Data are normalized to renilla luciferase activity and shown relative to C1-scr cells. (PDF 107 KB) [file 40170_2014_142_MOESM8_ESM.pdf]

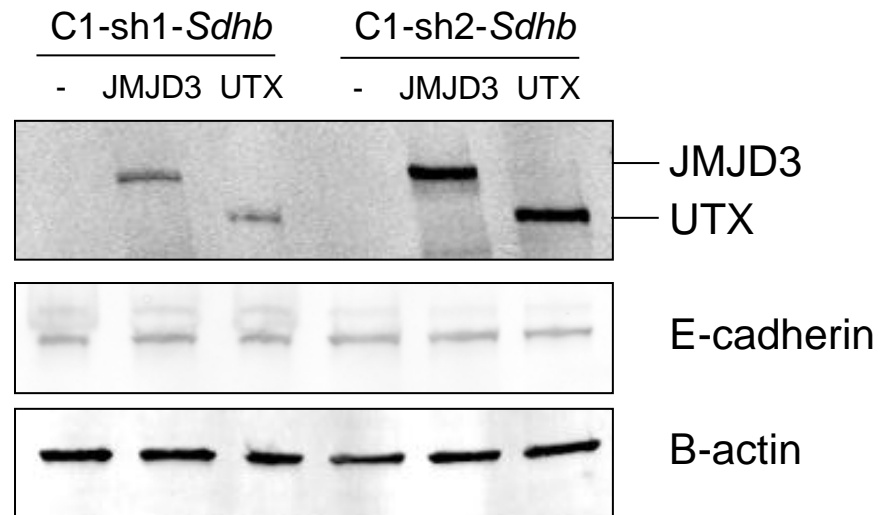

Supplement: Supplementary file 9 — Additional file 9: Figure S6: Overexpression of H3K27 demethylases, JMJD3 and UTX, does not promote MET in Sdhb knockdown cells. C1-sh1-Sdhb and C1-sh2-Sdhb cells were transfected with HA-tagged JMJD3, UTX, or GFP constructs. Lysates were collected 7 days post-transfection. Western blot detection of HA-tagged JMJD3 and UTX, E-cadherin, and β-actin. (PDF 67 KB) [file 40170_2014_142_MOESM9_ESM.pdf]

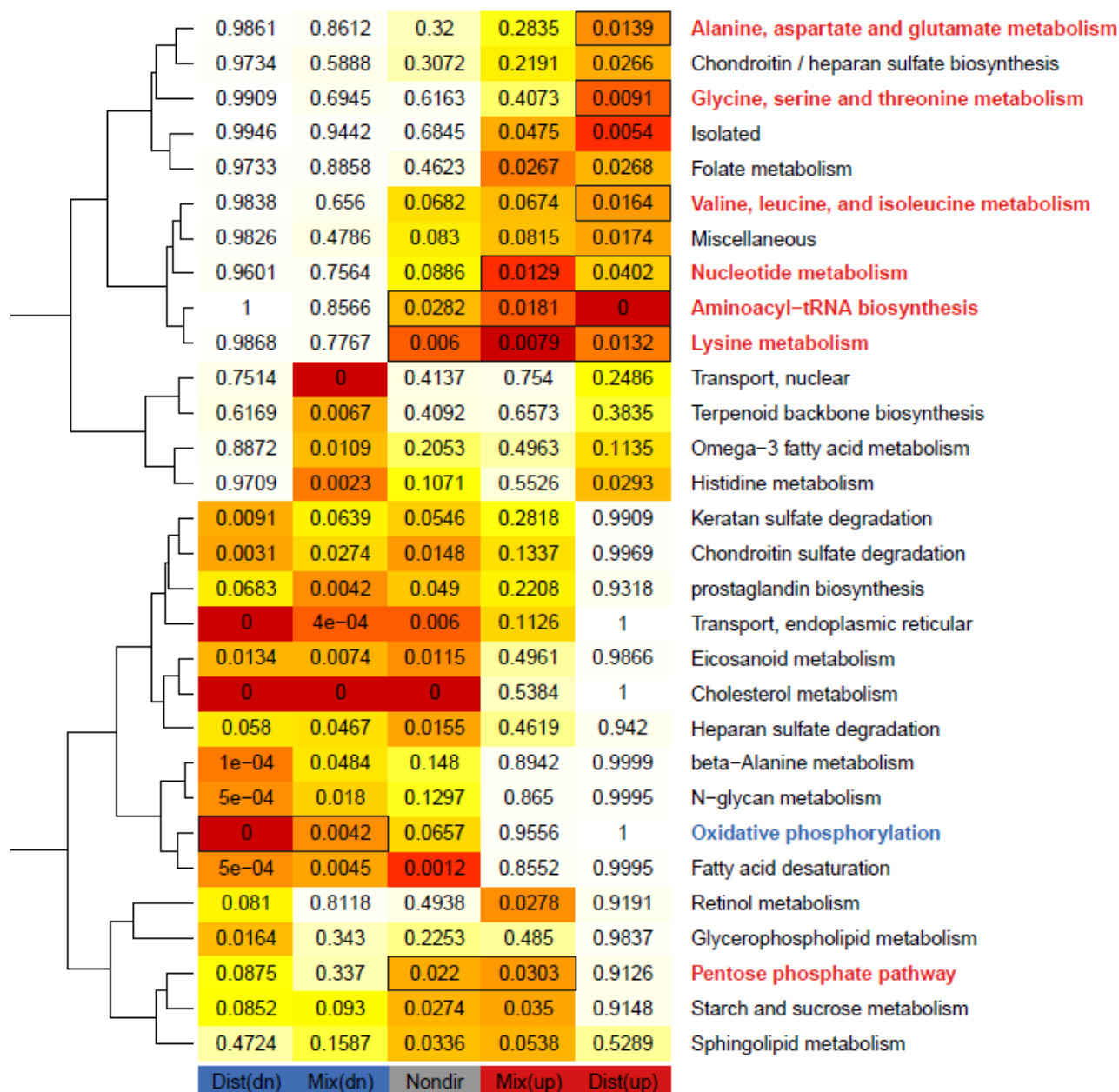

Supplement: Supplementary file 10 — Additional file 10: Figure S7: Sdhb knockdown affects the transcriptional regulation of the metabolism of amino acids, nucleotides, and oxidative phosphorylation. In order to identify metabolic pathways influenced by transcriptional regulation, we performed a gene set analysis using the pathways defined by the human genome-scale metabolic network HMR2. The resulting heat map shows the top significant pathways affected by transcriptional changes. The leftmost and rightmost columns show p values for the pathways being coordinately regulated in a distinct direction. The second columns from the left and right show the p values for pathways having a significant subset of upregulated or downregulated genes. The middle column shows the p values for pathways affected by transcriptional regulation in general, regardless of the direction. The pentose phosphate pathway, providing backbones for both nucleotides and amino acids, is upregulated. This pattern is also seen for pathways related to nucleotide metabolism and amino acid metabolism (marked in red). Oxidative phosphorylation is significantly downregulated (marked in blue). For the relevant pathways, p values <0.04 are marked with a black box. (PDF 100 KB) [file 40170_2014_142_MOESM10_ESM.pdf]
